# Characterization and modeling of the serum concentration of osteocalcin in breeding sows and its interaction with biochemical indicators: A review

**DOI:** 10.5455/javar.2022.i633

**Published:** 2022-12-31

**Authors:** Gerardo Ordaz, José Antonio Rentería, Gerardo Mariscal

**Affiliations:** Centro Nacional de Investigación Disciplinaria en Fisiología y Mejoramiento Animal-INIFAP, Querétaro, México

**Keywords:** Breeding sows, osteocalcin, energy metabolism

## Abstract

Adipose, muscle, and bone tissues modulate the metabolic state of mammals. However, the role of bone tissue as a metabolic state modulator in sows has not been studied. During the gestation–lactation transition, sows undergo metabolic adaptations to meet their nutritional requirements. Among these adaptations, bone remodeling is characterized by the synthesis and inhibition of hormones that participate, together with hormones from other tissues, in fetal development and lactogenesis. Osteocalcin is a hormone synthesized by the bone tissue which has been associated in different biological models with the improvement of the metabolic state. However, in sows, published results on the concentration of osteocalcin are scarce, and its concentration throughout the reproductive cycle is unknown. Therefore, with information from published trials on the measurement of serum osteocalcin, a structured review was conducted under the following objectives: (1) to review the promising effect of osteocalcin on energy metabolism in different models and (2) to characterize and model the serum concentrations of osteocalcin during the reproductive cycle of the sow. According to the review, the results obtained for humans and other animal models suggest that osteocalcin regulates energy metabolism, which has been associated with the need for integrated metabolism to cope with the metabolic demand during gestation and lactation in mammals. If these effects are significant in the sow, current recommendations for dietary balance should be reconsidered, particularly during the gestation–lactation transition period. According to mathematical modeling, it was the period in which the lowest concentration of osteocalcin was found.

## Introduction

The adoption of recent technologies has resulted in substantial improvements in sow productivity in recent decades [1]. In the 1980s, the first swine production systems were developed to intensify the rearing of this species. However, the productive potential of sows was not optimal, which was reflected in the high production costs [2]. The development of hyperprolific sows was initiated as a solution to the low productivity problem through the implementation of genetic improvement programs during the 90s [3–5]. However, the increase and rapid dissemination of this type of sow globally translated into difficulties, rather than benefits. The genetic improvement of reproductive indicators was not concomitant with the knowledge of the physiology of this new type of sow, which caused productivity gaps in the systems [6].

Currently, there is still a productivity gap in the systems, which is associated with the fact that modern sows face considerable metabolic challenges. During the transition between gestation and lactation, the sow must resort to metabolic modifications because of the increased demand for nutrients for fetal development and lactogenesis [7]. These metabolic adaptations lead to a deficit in feed intake at lactation, generating a catabolic state in sows owing to the mobilization of their body reserves, with adipose, muscle, and bone tissues being affected to a greater extent [7,8]. The deficit in feed intake of lactating sows is mainly compensated by the catabolism of body reserves [8]. During lactation, a sow’s energy metabolism is affected both by their body condition at the end of gestation and by their energy intake [9,10]. In most cases, the alteration of fat metabolism is accompanied by the catabolism of muscle tissue and changes in protein metabolism. Low energy intake implies reduced protein availability and subsequent alterations in biochemical indicators [11,12].

A third important reserve tissue is the bone. The skeleton of the sow is the main source of calcium during pregnancy and lactation [13]. Bone tissue turnover increases during the peripartum period because of calcium demand for fetal skeletal formation, uterine contractions, and early lactation [14]. However, as serum calcium levels stabilize during the reproductive cycle of sows [13–15], bone markers are useful alternatives for monitoring the bone metabolism of sows [15]. As a marker for bone formation, osteocalcin can provide information on bone turnover, calcium metabolism, and thus the metabolic status of the sow [16]. It has been hypothesized that energy metabolism, reproduction, and bone mass may have a common hormonal regulatory mechanism [17]. This conjecture is associated with insulin and leptin [18] acting on osteoblasts to stimulate or inhibit osteocalcin, a hormone that modulates insulin sensitivity [19]. However, there is little evidence of the behavior of osteocalcin concentration throughout the reproductive cycle of the sow, and no information on its effect on the energy metabolism of the sow. Therefore, the objectives of this study were (1) to review the promising effect of osteocalcin on energy metabolism in different models, and (2) to characterize and model the serum concentrations of osteocalcin during the reproductive cycle of the sow.

## Methodological Approach

Because the data were obtained from existing data, no approval was applied for from an animal care and use committee.

### Schematic modeling

Information from the main research on this topic was used for the characterization and schematic modeling of serum osteocalcin concentration and its relationship with energy metabolism in different biological models. The information was analyzed under the methodological approach of the General Systems Theory, which postulates that, with the integration of different scientific disciplines, the solution of problems is achieved integrally [20]. In the “real world”, complex scenarios and processes cannot be classified by their correspondence with a single discipline, resulting in complex systems [21,22]. A complex system represents a slice of reality, conceptualized as an organized whole, in which the elements are characterized by 1) not being separable, 2) having a specific delimitation (feedback), and 3) not being studied in isolation [23,24]. To study a phenomenon in isolation is to eliminate the analysis of the context [environment] in which observable relationships develop, which is neither ideal nor possible. Every biological system interacts directly with the environment [23].

Complex systems, such as swine production systems, are generally composed of four elements: context, humans, animals, and technology [24]. However, for this review, the technological component was prioritized because a production system of this nature is determined by its technical elements. This is represented in two contexts: 1) the physical context associated with alternatives used to control variability, and 2) the biological context associated with the knowledge generated to control the parameters inherent to the biology of the species [25]. In addition, the optimal balance of the four components to reduce variability to zero is not possible because a system in total equilibrium runs the risk of disappearing due to the precision exerted by a greater entropy contained in its products; therefore, biological systems move away from equilibrium for as long as possible. Therefore, for a system not to enter entropy, it must: (1) invest in the process of increasing the amounts of energy extracted from the environment by modifying the biological system [sow] through technology and (2) transfer the price of energy loss to the subsystems by modifying the interaction between the system’s components [22].

Hence, the serum concentration of osteocalcin in breeding sows was characterized and modeled using two schematic organization models. The first model contained a black box approach, where the factors attributable and not attributable to the phenomenon (and which were likely to condition the operation of the system) were obtained. In the second model, a more formal approach was considered. The data were analyzed considering Goodall’s [26] criteria: 1) internal homogeneity concerning properties of the system; 2) relative interdependence of the components of the system; and 3) related disciplines as a basis for breaking down the system. This eliminated, to the greatest possible extent, any inconsistencies that might bias the perception of reality.

## Mathematical Modeling

### Database description

A database containing indicators of the characteristics of the animals used, diet composition, and serum concentrations of osteocalcin, calcium, and phosphorus during gestation and lactation was developed. Information was collected from 12 articles published in scientific journals indexed in PubMed and Science Direct. Studies that met the following criteria were included: 1) adequately described research methods (feeding, sampling, and blood analysis); 2) the experiments always had a control group. Because of the limited results that the search yielded (17 publications) for the measurement of osteocalcin in reproductive sows, the control group of each selected experiment was used to characterize the osteocalcin concentration in serum during gestation and lactation; 3) the genotypes used in the research were related to the genotypes used in current swine production systems; studies using Guinea pigs and Vietnamese pigs used in research as biological models were omitted; and 4) serum osteocalcin, calcium, and phosphorus concentrations were determined at least during three different points in the reproductive cycle of the sow.

The main variables in the database about animals, i.e., their dietary composition and the serum concentrations of osteocalcin, calcium, and phosphorus, are reported in Table 1. The response variables (dependent) were the serum concentrations of osteocalcin (ng/ml), calcium, and phosphorus (mmol/l). The values of all variables for each observation could not be determined for all the observations. Therefore, the number of observations used for statistical analysis differed between the response variables.

### Statistical analysis

The data were analyzed according to the method of St-Pierre [27], who considered the random effect of a study and its possible interaction with fixed-effect factors. The MIXED procedure (SAS Institute. Inc., Cary, NC) was used to solve the following base model:

*Y_ij_* = *b*_o_ = *b*_1_*D* + *b*_2_*D*^2^ + *b*_3_*D*^3^ + *s_i_* = *a_i_D* + *e_ij_*, (1)

where, *Y*_ij_ is the observed result for the dependent variable (*Y*) in the *i*th experiment according to evaluation day (*D*), *i* = 1, 2,…, 143 (1, 2, …, 115 = gestation phase; 116, 117, …, 143 = lactation phase); b_0_ is a general intercept; *D* is the evaluation day; *b*_1_, *b*_2_, and *b*_3_ are the regression coefficients for *D, D*2*,* and* D*3 (fixed effects); is the random effect of the study (i.e., a change in the intercept for each study); *s_i_* is the random interaction of the study × *D* (i.e., a change in the linear term for each study); and is *e_ij_* the residual error.

In Equation (1), it is assumed that


$$S_{i}\approx N(0,\;\sigma_{s}^{2}\mathrm{),}a_{i}\approx N(0,\;\sigma_{s}^{2}\mathrm{),}$$

$$e_{\mathrm{ij}}\approx N(0,\;\sigma_{s}^{2}\mathrm{),}\;y\;\mathrm{Cov}(s_{i},\;e_{\mathrm{ij}})\;=\;0.$$

As the data were drawn from various investigations, each with its own experimental design, it was important to properly estimate the observations (the reported means) according to their relative precision (SE). Therefore, observations were weighted by the number of animals in each trial to account for the uneven residual variation between trials [28]. An unstructured variance-covariance matrix (TYPE = UN in the MIXED procedure) was used to model the random intercepts and slopes, allowing for random covariance between the slopes and intercepts across randomized studies [27].

**Table 1.** Descriptive statistics of the indicators included in the data set.

Indicator	Trials number	Obs. number	Average	SEM	Min.	Max.
Sows number/trial	12	92	26.7	3.03	5.0	100.0
Diet composition						
Gestation						
Crude protein, %	12	84	14.5	0.13	13.0	15.1
Metabolizable energy, kcal/kg	12	84	3,115.9	17.5	2,772.0	3,230.0
Calcium, %	12	84	0.83	0.02	0.70	1.0
Phosphorus, %	12	84	0.56	0.01	0.50	0.66
Lactation						
Crude protein, %	12	84	18.1	0.15	16.5	21.0
Metabolizable energy, kcal/kg	12	84	3,359.1	8.6	3,280.0	3,470.0
Calcium, %	12	84	0.95	0.02	0.71	1.20
Phosphorus, %	12	84	0.62	0.01	0.54	0.69
						
Osteocalcin, ng/ml	12	92	90.2	3.9	28.5	188.3
Calcium, mmol/l	12	92	1.95	0.06	1.2	3.2
Phosphorus, mmol/l	12	92	2.05	0.04	0.72	3.3

**Figure 1.**
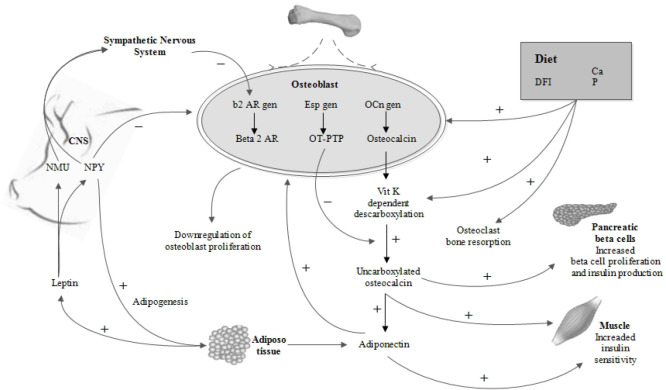
Relations between the skeleton and energy metabolism. Figure modified from Wolf [32].

### Interaction of osteocalcin with metabolic indicators in different biological models

Over the past few decades, several studies, mainly in mice, have been published [29,30] that provide evidence for osteocalcin-driven metabolic effects. Such studies have focused on the question of why a bone-specific hormone would regulate energy metabolism. As reported on the role of the skeleton in energy metabolism in murine models, when translated to reproductive sows, osteocalcin in sows reflects an essential need to integrate homeorhetic changes, i.e., “coordinated changes in the metabolism of tissues body tissues necessary to support a physiological state” [31]. By understanding and modulating the concentration of osteocalcin and its effects on biochemical indicators in reproductive sows, it can be possible to implement feeding strategies that favorably modulate the metabolic state and increase productivity. However, this also requires an understanding of the interactions between skeleton, energy, and protein metabolism and their effects on metabolism in general (Fig. 1).

### What is known about osteocalcin as a modulator of energy metabolism?

When it was discovered that obesity lowers the incidence of osteoporosis in humans, the idea that the skeleton may play a role in energy homeostasis and homeorhesis was initially put forth in 1993 [33]. The relationship between bone metabolism and glucose in knockout mice was first reported in 1996, both in normal mice and in mice specific for osteoblasts that encode osteotesticular protein tyrosine phosphatase, a protein that has an affinity to be expressed in bone, testis, and ovary [34,35]. Both Esp knockout animals displayed lower blood glucose levels, higher serum insulin concentrations, improved glucose and insulin tolerance tests, larger pancreatic islets, and greater cell proliferation in addition to higher insulin sensitivity and lower visceral fat.

A hormonal and neurological mechanism that controls bone remodeling was established in 1998 by Corral et al. [36]. Leptin improves the osteoblastic development of bone marrow progenitors and prevents late differentiation to adipocytes, according to a 1999 publication by Thomas et al. [37]. Therefore, it is hypothesized that leptin may function largely in the maturation of stromal cells in both lineages to regulate these two differentiation routes physiologically, the idea that the same hormones control both bone and energy metabolism dates back to 2000 [38]. By proving that leptin, via a hypothalamic pathway, is the primary regulator in the suppression of bone development, *in vivo* evidence of central regulation of bone mass is provided.

Leptin influences energy balance and the neuroendocrine axis through hypothalamic linkages, according to Ahima [39], who published her findings in 2000. In 2002, Cornish et al. [40] published the first study demonstrating the systemic effects of leptin administration on histomorphometry and resistance in bone tissue in wild mice. They found that leptin: (1) directly regulates bone cell function *in vivo *and decreases bone fragility, and (2) the peripheral effect of leptin (mediated by insulin) outweighs the central effect.

In 2004, it was demonstrated that excessive bone mass in fat-free mice could be rectified with the infusion of transgenic leptin, demonstrating that it is responsible for the bone phenotype [41]. This demonstrated that the integrity of sympathetic transmission is required for the increase in bone resorption induced by gonadal failure and contributed to the discovery in 2005 that leptin controls, through a neurological mechanism, the two components of bone remodeling. The receptor activator nuclear factor-kB stimulates bone resorption in the sympathetic nervous system (SNS; RANKL). The phosphorylation of ATF4 is necessary for this sympathetic activity. Due to Cocaine and Amphetamine Regulatory Transcript (CART) whose expression is regulated by leptin, and which was repressed in the model (ob/ob), which prevents bone resorption by modifying RANKL, bone resorption does not rise in gonadectomized mice lacking the B2 adrenergic receptor receptor [42].

It was not until 2007 that Lee et al. [29] established that osteocalcin, which is a bone hormone, regulates energy metabolism and glucose homeostasis. In 2009, for the first time in humans, plasma osteocalcin was reported to be inversely related to fat mass and plasma glucose [43]. In addition, the association of osteocalcin with insulin sensitivity and metabolic syndrome has been reported [44,45]. It should be noted that since the proportion of carboxylated osteocalcin (Gla) was reduced in *Esp*-/-mice, it was suggested that *Esp* regulates the carboxylation of osteocalcin and that non-carboxylated osteocalcin (Glu) is the one that participates as a hormone that regulates glucose metabolism [29]. Furthermore, *Esp *was found to inhibit insulin signaling in osteoblasts by dephosphorylating the insulin receptor, and insulin signaling inhibited *FoxO*, which induced *Opg* and *Esp *expression but reduced osteocalcin expression [46,47]. Because reduced *Opg* expression leads to increased bone resorption, which induces osteocalcin decarboxylation, it was proposed that serum osteocalcin Glu, by inducing bone resorption, increases insulin signaling in osteoblasts [46].

### Leptin as a modulator of osteocalcin

Since leptin was discovered in 1994, the majority of studies on it have focused on how it affects calorie intake, reproductive function, and appetite regulation [48]. Clinical studies, however, have demonstrated recently that leptin also impacts bone remodeling [17]. Based on the unique properties of this hormone for appetite, reproduction, or energy expenditure in vertebrates, these investigations [30,39,49] were conducted. For instance, worms and flies can have poor nutritional status, be fertile or infertile, and still not produce leptin. Leptin is now considered to be typical of vertebrates; in fact, it first appeared with the development of bone [30]. It suggests that leptin may co-regulate the metabolism of both bones and energy.

**Figure 2.**
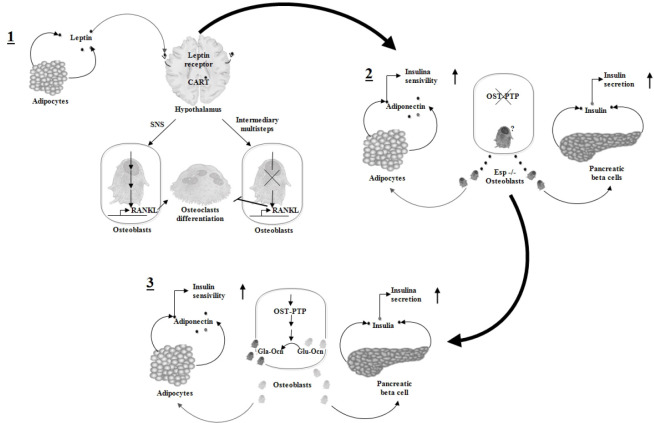
This figure, modified by Lee and Karsenty [17], schematically represents the regulation of bone mass by fat. 1) Leptin binds to its receptor on hypothalamic neurons, subsequently via the SNS via CART expression of RANKL in osteoblasts. 2) Osteoblasts regulate insulin gene expression in β cells and adiponectin gene expression in adipocytes. Osteoblasts or *Esp* -/- were co-cultured with β cells or adipocytes. This procedure resulted in increased insulin gene expression, increased insulin secretion, and increased adiponectin gene expression, which resulted in increased insulin sensitivity. 3) Osteocalcin in its non-carboxylated form (Glu-OC) derived from osteoblasts improves glucose kinetics. OST-PTP, the product of the *Esp* gene, favors, through still unknown mechanisms, the carboxylation of osteocalcin. In the absence of *Esp*, most osteocalcin is not carboxylated. This non-carboxylated form of osteocalcin increases insulin gene expression in β cells and adiponectin gene expression in adipocytes, resulting in increased insulin secretion and insulin sensitivity, respectively.

Leptin has been shown to affect bone metabolism in a variety of ways. Studies in mice have shown that leptin gene-mutated ob/ob mice have a high bone mass phenotype similar to leptin receptor-deficient db/db mice [38]. Leptin gene therapy corrects bone abnormalities in ob/ob mice [50], thus indicating that leptin is responsible for the particular bone phenotype. In 1995, it was reported that the medial basal hypothalamus had the highest density of neurons expressing the leptin receptor and was a critical target of leptin [51]. In leptin-deficient mice, leptin treatment has been shown to cause bone loss, and it has been determined that leptin suppresses bone production via the hypothalamic relay [38]. Additionally, leptin has been demonstrated to control bone formation via the SNS (Fig. 2). This finding suggests that the ventromedial hypothalamus’s neurons are implicated in leptin’s anti-osteogenic action [52]. As a link between the hypothalamus and the bone, this action also needs a healthy SNS. Functional β2 adrenergic receptors were also found in osteoblasts and β2 adrenergic antagonist could increase bone mass in mice [52]. This proves that signals are sent to osteoblasts via the SNS after leptin attaches to its receptor on ventromedial neurons [30].

Peripheral leptin enhances bone mass by interacting with mesenchymal stem cells in the bone marrow, as well as with osteoblasts, osteoclasts, and chondrocytes, in addition to its effects on bone metabolism through the central pathway [53,54]. High-affinity leptin receptors have been found in the bone marrow of humans [37], where they can differentiate into adipocytes or osteoblasts. Leptin has also been shown to enhance proliferation and mesenchymal stem cell differentiation in the osteoblastic lineage while inhibiting those in the adipocyte lineage [55,56]. Leptin can promote osteoblast proliferation, *de novo* collagen synthesis, and mineralization *in vitro*, and facilitate osteoblast signaling to osteoclasts [57].

In summary, leptin affects bone metabolism through the central and peripheral pathways. On one hand, when acting via the central pathway, leptin appears to inhibit bone formation and promote bone resorption, thus leading to bone loss. In contrast, via the peripheral pathway, leptin appears to increase bone formation and inhibit bone resorption, thus increasing bone mass.

### Neuropeptide Y (NPY) as a modulator of bone metabolism

NPY, a 36-amino acid peptide, is highly expressed in the arcuate nucleus of the hypothalamus [58]. NPY infusion led to a significant reduction in cancellous bone volume in mice [38]. Studies in mice have reported that NPY-deficient animals appear to have increased bone mass associated with increased osteoblast activity [59]. Brain-specific NPY overexpression and Y-receptor deactivation models have also been reported, thereby revealing a potent anabolic pathway of the NPY system in the control of bone metabolism [60]. Moreover, central leptin signaling can also inhibit NPY production in leptin-sensitive NPY neurons, suggesting a possible interaction between NPY and leptin in the regulation of bone formation [54].

### Vitamin K as a cofactor for osteocalcin carboxylation

The first reports indicating the effect of vitamin K on bone metabolism were published in the 1970s after malformations were observed in infants born to women treated with vitamin K antagonist drugs [61]. Numerous studies have reported that vitamin K deficiency in diet or circulation is associated with lower bone density [62–64]. Vitamin K has also been shown to affect osteocalcin carboxylation [65], reduce urinary calcium excretion, and improve bone turnover profile [66].

For osteocalcin to exert an effect on energy metabolism, it must be decarboxylated; however, there is no extracellular or circulating gamma decarboxylase, which suggests that this process depends on bone resorption. This hypothesis was derived from the following observations: 1) Gla-osteocalcin binds to the mineralized matrix through Gla residues, and can be released from the matrix by osteoclasts [67]; 2) Gla-osteocalcin residue Gla can be decarboxylated when exposed to an acidic pH, and bone resorption causes acidification of the bone matrix [68], and 3) vitamin K functions as a cofactor for the enzyme glutamate carboxylase (GGCX) [69].

**Figure 3.**
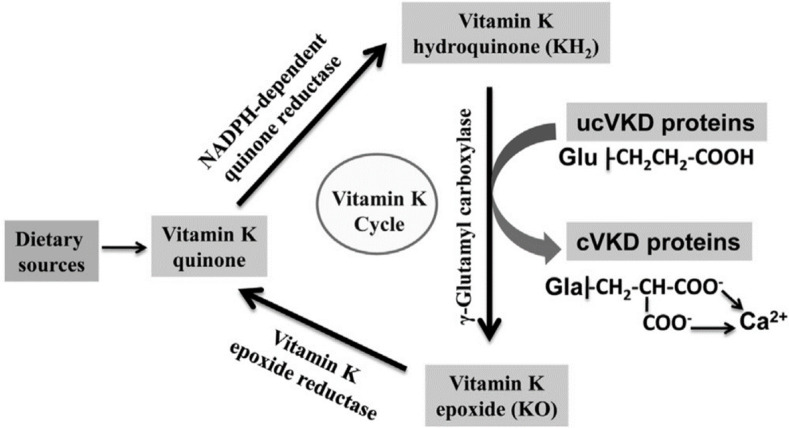
Vitamin K cycle. Vitamin K functions as a cofactor for the GGCX, which is essential for the conversion of glutamate (Glu) to g-carboxyglutamate (Gla) residues of G-dependent proteins. Vitamin K (VKD): the g-carboxylation transforms the sub-carboxylated VKD proteins (ucVKD) into carboxylated ones (cVKD). Source: Manna and Kalita [70].

Therefore, gamma-carboxylation (Fig. 3) is dependent on vitamin K and is essential for the protein to have a high affinity for minerals and to allow osteocalcin (Gla-osteocalcin) to attract calcium ions and incorporate them into hydroxyapatite crystals [71]. In contrast, Gluosteocalcin has a lower affinity for bone and is found in greater proportions in the circulation [72]. Therefore, carboxylation or its absence favors the release of osteocalcin into circulation, respectively. It has been shown *in vitro* in isolated islets and primary adipocytes that the carboxylate form is inactive, and the non-carboxylate form is active in isolated islets and primary adipocytes [17]. This was corroborated by the exposure of adipocytes to carboxylated (Gla-) and non-carboxylated (Glu-) recombinant osteocalcin. Adipocytes exposed to Glu-osteocalcin produced twice the amount of adiponectin. Similarly, culturing pancreatic cells cultured with Glu-osteocalc showed an increase in the amount of insulin (i.e., 1.5-fold) and the level of cyclin D (an indicator of cell proliferation, 2.5-fold), with a subsequent increase in pancreatic β-cells. Similarly, insulin sensitivity also increased, thus improving glucose kinetics [30].

However, the molecular mechanisms underlying the beneficial role of vitamin K in insulin sensitivity and glucose homeostasis remain unclear. At the general systemic level, the concentration of total osteocalcin includes the values of both Gla- and Glu-osteocalcin levels. The percentage of Glu-osteocalcin decreases in response to vitamin K supplementation and increases with vitamin K depletion [73]. Interestingly, vitamin K intake, which causes a decrease in Glu-osteocalcin concentration, has been reported to reduce insulin resistance in patients with diabetes [74,75], which is opposite to the expected outcomes from animal studies [17]. Therefore, there is an inconsistency between human evidence and experimental models regarding the involvement of osteocalcin in glucose metabolism.

### Characterization and modeling of the serum concentration of osteocalcin in breeding sows

The descriptive statistics for the evaluated indicators are presented in Table 1. An average of 26.7 sows per trial was used, but there was considerable variability in the number of sows used in the trials (ranging from 5 to 100 sows per trial). Serum osteocalcin concentrations ranged from 28.5 to 188.3 ng/ml, with a mean of 90.2 ng/ml across all studies. The mean serum calcium and phosphorus concentrations of the sows ranged from 1.2 to 3.2 and from 0.7 to 3.3 mmol/l, respectively.

**Figure 4.**
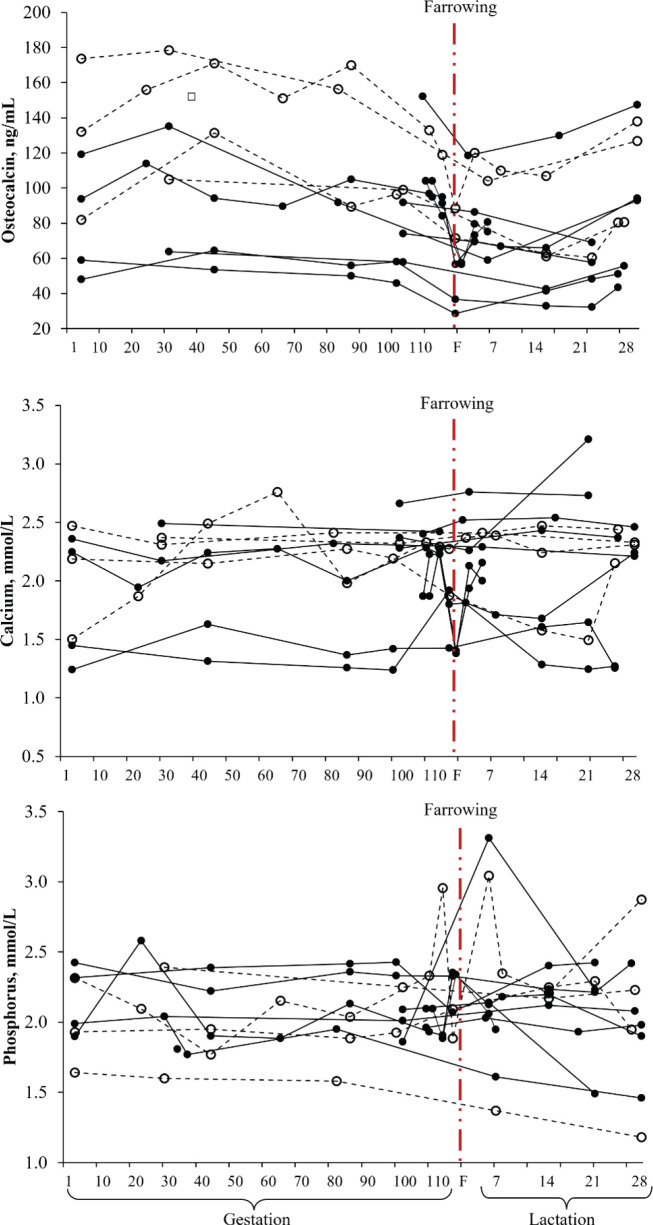
Effect of the sampling day (productive phase) on the serum concentration of osteocalcin, calcium, and phosphorus (12 trials, 588 sows evaluated: 222 primiparous ○ and 366 multiparous ●).

Figure 4 shows the uncorrected serum concentrations of osteocalcin, calcium, and phosphorus on each sampling day for each trial. The variation in the concentrations of serum osteocalcin, calcium, and phosphorus between trials is quite evident (Fig. 4). The estimated parameters, SE, root mean square error (RMSE), and parameters of the covariance components for the model [1] are presented in Table 2. The sampling day provided evidence of cubic behavior for the serum osteocalcin concentration, while serum calcium and phosphorus concentrations showed linear behavior (Table 2).

Regarding the linear behavior of serum calcium and phosphorus concentrations, breeding sows have higher nutrient requirements at the end of gestation and during lactation [7]. However, data on the mineral requirements, especially calcium and phosphorus, are limited. Inadequate nutrition, as well as suboptimal feed intake at times with increased nutritional demand, can lead to unbalanced mineral nutrition or even mineral deficiency, negatively affecting bone quality and its association with metabolic indicators [76,77].

**Table 2.** Prediction equations for linear or polynomial regression of the response of osteocalcin, calcium, and phosphorus according to the day of sampling and type of sow.

		Parameters				Covariance components ^a^		
Dependent variable	Independent variable	Estimator	SE	*p-*value	RMSE	σ_s_^2^	σ_a_^2^	σ^2^_s,a_
Osteocalcin, ng/ml	Intercept	15.8798	13.96	0.0046	23.31	0.9707	108.37	0.0312
	*D*	1.1203	0.56	0.0186				
	*D* ^2^	−0.0211	0.01	0.0271				
	*D* ^3^	0.0001	0.00004	<0.0001				
Calcium, mmol/l	Intercept	0.9186	0.31	0.0003	0.341	0.6537	0.8169	0.0147
	*D*	−0.0018	0.0002	0.0062				
Phosphorus, mmol/l	Intercept	0.2085	0.07	0.0004	0.538	0.898	0.894	0.0094^b^
	*D*	−0.0003	0.0001	0.0145				
Primiparous sows								
Osteocalcin, ng/ml	Intercept	61.9401	20.71	0.0062	21.97	0.5670	83.283^b^	0.0016
	*D*	1.7522	0.82	0.0432				
	*D* ^2^	−0.0287	0.01	0.0382				
	*D* ^3^	0.0001	0.00006	0.0491				
Multiparous sows								
Osteocalcin, ng/ml	Intercept	−6.0179	13.03	0.0215	18.97	1.191	130.207	0.0026
	*D*	0.6059	0.62	0.0435				
	*D* ^2^	−0.0130	0.01	0.0136				
	*D* ^3^	0.00006	0.00004	0.0541				

a σ_s_^2^ is the variance estimate for the intercept due to trial; σ_a_^2^ is the estimated variance for the random linear term for sampling day (i.e., trial × sampling day interaction); σ_2__s,a_ is the estimated covariance between the intercept and the slope.

Estimated variance or covariance was not significantly different from zero (*p* > 0.05).

In breeding sows, due to the strong correlation that exists between calcium and phosphorus regulation, the serum concentrations of these minerals are constant. This characteristic is observed in the prediction models (Table 2), which makes it difficult to deduce whether there is a mineral deficiency. Therefore, indicators of bone resorption, such as serum osteocalcin, indirectly predict the degree of bone remodeling, the metabolic status of the reproductive sow, and whether there is mineral use of calcium and phosphorus in the bone matrix [15].

**Figure 5.**
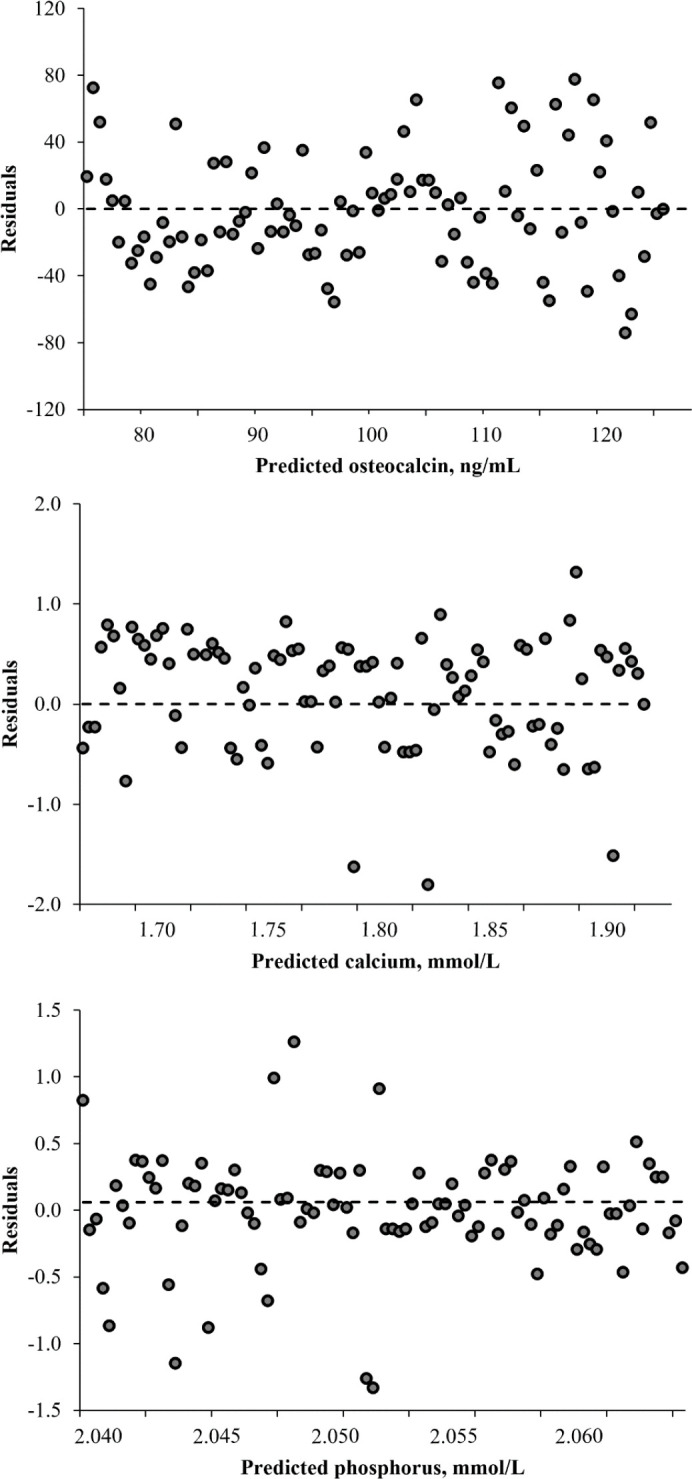
Plot of residuals (observed – predicted) against osteocalcin, calcium, and phosphorus predicted from the mixed model. The line represents the regression of the residuals on predicted osteocalcin [*Y *= 0.000083 (23.69) – 8.8 × 10^-7^ (0.26) × predicted osteocalcin; *R*^2 ^= 0.004; *p *> 0.05], predicted calcium [*Y* = 0.00028 (3.79) – 0.00016 (2.08) × predicted calcium; *R*^2 ^= 0.001; *p* > 0.05] and predicted phosphorous [*Y* = 0.0623 (11.84) – 0.0303 (5.77) × predicted phosphorus; *R*^2 ^= 0.001; *p *> 0.05].

**Figure 6.**
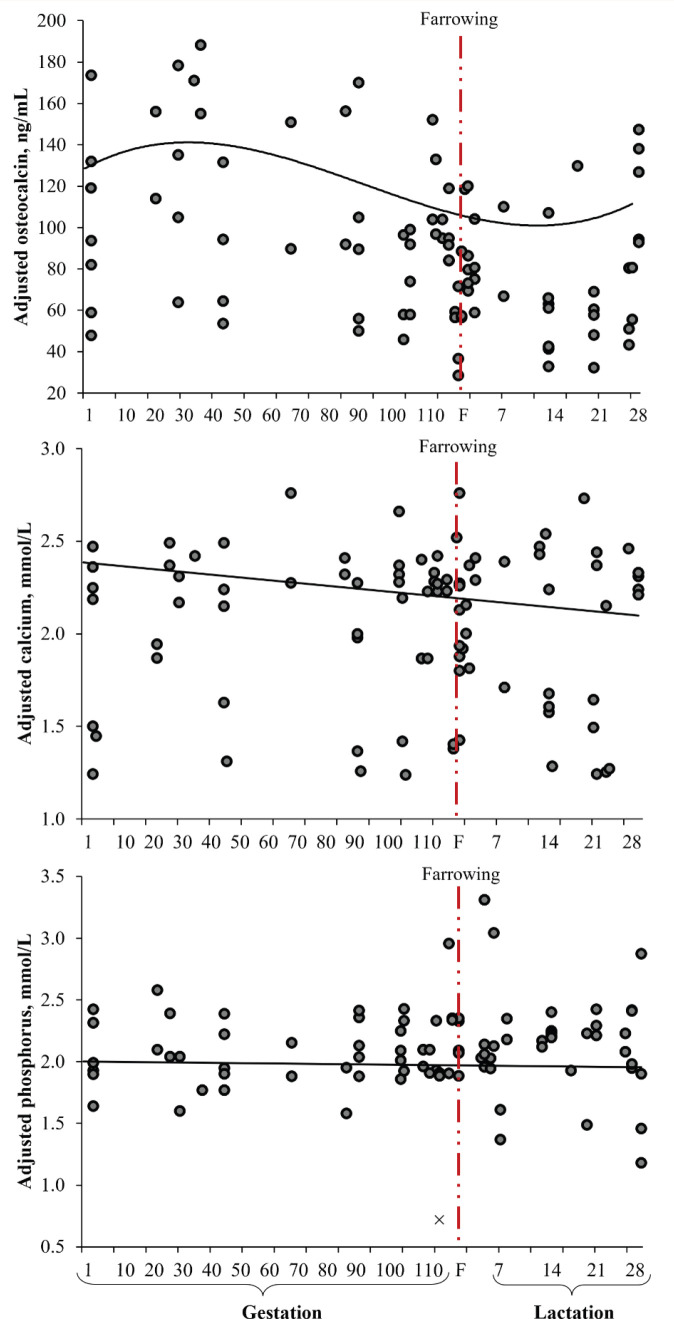
The average concentration of osteocalcin, calcium, and phosphorus versus prediction model (solid line) in response to gestation and lactation period [solid line calculated using Equation (1)].

In terms of the distribution of the residuals, no evidence of bias (linear or nonlinear) was found in the prediction models for serum concentrations of osteocalcin, calcium, and phosphorus (Fig. 5). Most residuals were less than 30 ng/ml for osteocalcin, while for calcium and phosphorus they were less than 0.5 mmol/l, which is equivalent to <33% of the average of each indicator. A strong relationship was observed between the adjusted and measured values of osteocalcin (adjusted *R* = 0.84), calcium (adjusted *R* = 0.87), and phosphorus (adjusted *R* = 91) (Fig. 6). This relationship indicates that the observations within the study were highly predictable.

The adjusted values of serum osteocalcin, calcium, and phosphorus levels were the observed values corrected for a study effect. However, the random effects of the trial [i.e., the variance due to the trials, the intercept (σ_s_^2^), and the linear effect of the sampling day (σ_s_^2^)] were large and differed significantly from zero (Table 2). This implies that both parameters in the cubic function (for osteocalcin) and the linear function (for calcium and phosphorus) depend on specific factors within each trial. This quantitative analysis generated more precise prediction models to explain the serum concentrations of osteocalcin, calcium, and phosphorus observed in each assay. However, predictions of future results may not be highly accurate with the models reported in Table 2 because the context for the development of future trials might differ [21]. In addition, important unidentified factors (other than those reported in trials already published and considered in the model) can be suggested that have an impact on the relationship between the environment, the day of sampling, and the serum concentrations of osteocalcin, calcium, and phosphorus.

**Figure 7.**
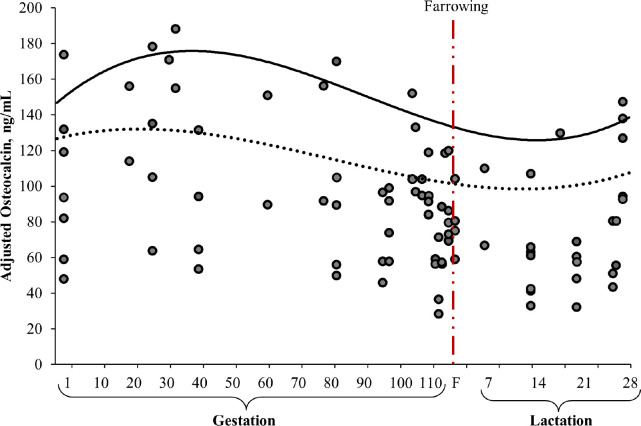
The average concentration of osteocalcin versus prediction model (solid line) in response to the gestation and lactation period according to the type of sow [primiparous; multiparous; solid line calculated using Equation (1)].

The age of the sow was one of the indicators evaluated that inherently modified the serum concentration of osteocalcin (Table 2 and Fig. 7). The day of sampling had the greatest impact on the serum concentration of osteocalcin because it established the productive stage (gestation, peripartum, or lactation) of the sows (Fig. 6). The age of the sows (classified as primiparous or multiparous sows) also affected the serum osteocalcin concentration, with primiparous sows showing higher concentrations (Table 2 and Fig. 7). In terms of the day of evaluation of serum osteocalcin concentration, on the farrowing day, bone deposition activity (concentration) was lower than that observed during pregnancy (Figs. 6 and 7). This favored physiological changes in the mineral mobilization from the maternal skeleton, parallel to the change in placental calcium transfer during gestation toward the production of calcium-rich milk [78]. This behavior reflects greater requirements for the skeletal development of fetuses and milk production [15,79]. Sows have been reported to deposit approximately 50% (10.4 ± 1.3 gm/piglet; 131 ± 12 gm/litter) of the calcium accumulated during the last 2 weeks of gestation [80].

According to the prediction models for serum osteocalcin (Table 2), it can be deduced that bone deposition evolves to a state of net loss during late gestation and lactation (gestation days 84–21). This is true even though this productive phase presents greater absorption efficiency. Therefore, it can be deduced that the minerals absorbed from the feed may not meet the higher calcium and phosphorus demands for fetal development and milk production or that feed intake is inadequate for the purpose described above [15,81]. It has been established that vitamin and mineral intake are also important factors associated with bone metabolism [77,82]. This is the case for vitamin D; 1,25-(OH)2D3 is involved in the transport and mobilization of calcium and phosphorus [83,84], and it facilitates the skeletal mineralization of fetal growth during gestation and milk production during lactation [85].

The higher serum concentration of osteocalcin in primiparous sows (Fig. 7) is associated with their continuous growth during gestation compared to that in multiparous sows. Primiparous sows may require more nutrients for the growth and development of muscle and skeletal tissues than multiparous sows [86]. However, nutrient requirements for multiparous sows vary according to weight loss from the previous lactation period and concomitant nutrient deposition. However, dietary calcium and phosphorus do not influence the serum concentration of osteocalcin [15,16].

## Conclusion

The metabolic adaptations that a sow undergoes during late gestation and lactation (independent of the fact that these are physiological adaptations) limit the productive potential of the sow, as they affect her energy consumption during lactation. This is reflected in body catabolism and its subsequent effect on post-weaning productive indicators: weaning-estrus interval, repeated services, fertility, and prolificacy. Therefore, strategies should be developed to minimize this problem. Studies on the effect of osteocalcin on energy metabolism in various biological models project this hormone as a possible means to modulate the metabolic state of sows during late gestation and lactation. Manipulating energy metabolism and bone metabolism in a coordinated manner would affect productivity and may have the potential to modify the post-weaning productive gap. If these effects are shown to be significant for pig production, prevailing recommendations for dietary balance should be reconsidered, particularly during the nursery period and lactation. Therefore, it is necessary to quantitatively determine the importance of bone resorption for health, production, and reproductive performance.

Mathematical modeling elucidates the relationships between the serum concentrations of osteocalcin, calcium, and phosphorus and the day of sampling (stage of the sow). This model shows that there are factors, such as age, that can interact with the productive stage of the sow to affect the serum concentration of osteocalcin. However, future work is needed to characterize and model the serum concentration of osteocalcin in sows and to more accurately associate the behavior of this hormone with biochemical indicators. This may be even more important for the current genotypes of hyperprolific sows in the context of developing strategies that favorably modulate metabolic state and increase sow productivity. This finding is consistent with the results reported for other animal models on the effect of osteocalcin on the metabolism of lipids and carbohydrates.

***Obituary:*** The authors wish to express our posthumous recognition to Dr. José Antonio Rentería Flores, DVSc, who was well known for his lifelong dedication to his work and his research interests, as well as for his active participation in the development of this publication. May he rest in peace!

## List of Abbreviations

CART, Cocaine and amphetamine regulatory transcript; Gla, carboxylated osteocalcin; Glu, non-carboxylated osteocalcin; NPY, Neuropeptide Y; RANKL, receptor activator nuclear factor-kB; RMSE, root mean square error; SNS, sympathetic nervous system.
